# Cell Reprogramming With CRISPR/Cas9 Based Transcriptional Regulation Systems

**DOI:** 10.3389/fbioe.2020.00882

**Published:** 2020-07-28

**Authors:** Ksenia M. Shakirova, Viktoriia Y. Ovchinnikova, Erdem B. Dashinimaev

**Affiliations:** ^1^Faculty of Biology, Lomonosov Moscow State University, Moscow, Russia; ^2^Koltzov Institute of Developmental Biology, Russian Academy of Sciences, Moscow, Russia; ^3^Center for Precision Genome Editing and Genetic Technologies for Biomedicine, Pirogov Russian National Research Medical University, Moscow, Russia

**Keywords:** human cell reprogramming, CRISPR/Cas9, transactivator systems, regenerative medicine, genome screening, dCas9

## Abstract

The speed of reprogramming technologies evolution is rising dramatically in modern science. Both the scientific community and health workers depend on such developments due to the lack of safe autogenic cells and tissues for regenerative medicine, genome editing tools and reliable screening techniques. To perform experiments efficiently and to propel the fundamental science it is important to keep up with novel modifications and techniques that are being discovered almost weekly. One of them is CRISPR/Cas9 based genome and transcriptome editing. The aim of this article is to summarize currently existing CRISPR/Cas9 applications for cell reprogramming, mainly, to compare them with other non-CRISPR approaches and to highlight future perspectives and opportunities.

## Introduction

Data that has been accumulated over many years regarding the mechanisms of cell differentiation has enabled the development of cell reprogramming – a brand new strategy in biotechnology. The ability to roll back somatic cells to a pluripotent state or even to switch one somatic cell type directly into another (transdifferentiation) has become an important breakthrough in cell biology due to the broad applications from fundamental studies to regenerative medicine and the treatment of genetic disorders. Early reprogramming technologies, such as somatic cell nuclear transfer (SCNT), and cell fusion, first performed about 60 years ago, confirmed that the differentiated state of somatic cells can be reversed (Briggs and King, 1952; Köhler and Milstein, 1975). Although these technologies were suitable for a number of applications (Köhler and Milstein, 1975; Lee et al., 2016), they are still too stochastic and uncontrollable for the majority of modern reprogramming purposes. The next level in reprogramming was exogenic overexpression of the transcription factors (TFs) in somatic cells. This approach was used by Takahashi and Yamanaka (2006) in their famous experiment of reprogramming somatic cells into induced pluripotent stem cells (iPSCs). overexpression of TFs still remains the most common and efficient way to change a cell‘s fate. Nowadays a great variety of techniques exists to allow that change. One of them might be CRISPR/Cas9 – a genetic engineering tool based on a bacterial antiviral defense system (Hsu et al., 2014). This system has undergone many modifications that allow not only DNA editing but also regulation of gene expression in different ways – by activation, repression or even chromatin remodeling. The vast range of CRISPR/Cas9 applications in cell reprogramming makes it the most promising among molecular tools.

In this review we describe the evolution of genetic engineering tools for cell reprogramming, summarize modern techniques based on CRISPR/Cas9 technology and provide comparisons with other approaches in order to outline the pros and cons as well as to determine future perspectives of this ground-breaking technology.

## CRISPR/Cas9 Applications in Cell Reprogramming

**FIGURE 1:**
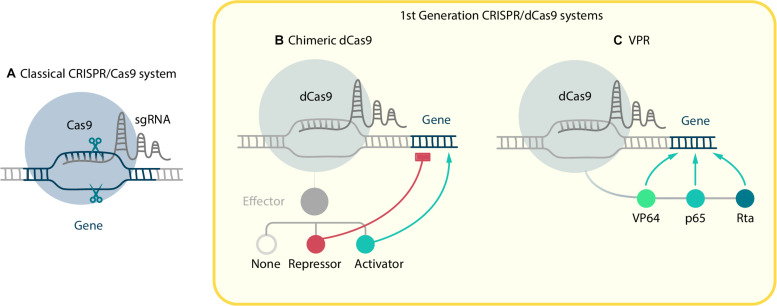
CRISPR/Cas9-based systems. **(A)** Classical CRISPR/Cas9 system, where sgRNA guided Cas9 performs a double-strand break in target locus. In order to manipulate gene expression the dCas9 mutant without nucleolytic activity was created. First generation CRISPR/dCas9 systems consist of two components: dCas9 fused with effector and sgRNA. They include **(B)** chimeric dCas9, which can physically block RNA polymerase and repress elongation or, if fused with effectors, activate or repress gene expression; and **(C)** VPR, where three activator domains VP64, p65, Rta are fused with dCas9.

The basic CRISPR/Cas9 gene editing system works like molecular scissors. It consists of two components: the RNA-dependent DNA endonuclease, Cas9, and small synthetic single guide RNAs (sgRNAs). Each particular sgRNA is designed to be complementary to a DNA-target region, so the Cas9-sgRNA complex binds exactly to a genome region that matches the sgRNA sequence and has a protospacer adjacent motif (PAM) nearby (Figure 1A). Then the Cas9 performs a double-strand break (DBS). The repair mechanism for such a DBS performs non-homologous end joining (NHEJ) and therefore usually introduces indel mutations, which result in reading frameshift and the generation of loss-of-function mutants. It makes CRISPR/Cas9 a convenient tool for genetic knockout.

It is a common scenario when, due to cross-antagonistic TFs interactions, cell fate depends on the prevalence of one of the TFs (Graf and Enver, 2009). For example, in PU.1:GATA1, antagonistic system dominance of PU.1 leads to cell differentiation into myeloid cells, while the dominance of GATA1 – to erythroid cells (Arinobu et al., 2007). Studies of the PU.1:GATA1 balance showed that the cell fate could be re-specified by ablation of agonistic TF (Galloway et al., 2005). This ablation can be performed by CRISPR/Cas9 mediated gene knockout. For example, CRISPR/Cas9 mediated knockout of *MyoD* promotes myoblast transdifferentiation into brown adipocytes (Wang C. et al., 2017). CRISPR/Cas9 mediated gene knockout can not only vary TFs translation, but also facilitates reprogramming by affecting cell proliferation and DNA methylation (Kaplun et al., 2019). Thus direct use of Cas9 nuclease is a convenient and easy-to-use tool for generating loss-of-function mutations that can contribute to cell reprogramming and transdifferentiation. Nevertheless, this approach alone is not applicable for reprogramming into pluripotent cells or transdifferentiation between distant cell types.

Numerous systems, based on modified Cas9, have been developed during the last decade. In order to expand CRISPR/Cas9 functions, Qi et al. (2013) created the dCas9 (dead Cas9) mutant, which is defective in nucleolytic activity but is still able to perform RNA-dependent DNA-binding. A chimeric dCas9 molecule, fused with any functionally active domain, can deliver effector-cargo to specific genome loci. It allows to perform precise manipulations with epigenetic regulation of expression in different ways, which is crucial for advanced cell reprogramming. The most commonly-used molecules that can be fused to dCas9 are transcription activators and repressors, epigenetic remodeling factors, reporters of expression, base editors, and even nucleolytic domains from other nucleases (Gilbert et al., 2013; Mali et al., 2013; Perez-Pinera et al., 2013; Tanenbaum et al., 2014; Tsai et al., 2014; Rees and Liu, 2018). The first two of these modifications are especially important for reprogramming purposes due to their ability to change the balance between different TFs. All the systems for CRISPR activation (CRISPRa) and CRISPR interference (CRISPRi) can be divided into two groups: first generation systems, in which a single effector domain is fused directly to the dCas9, and second generation systems, in which activation or repression is amplified due to recruiting multiple copies of effector. It is important that such activation or repression via dCas9 allows to control gene expression without directly interfering with the DNA, moreover, that effect can be reversible. The ability to change already altered gene expression is vital for some cell reprogramming experiments, for example, if it is necessary to reprogram somatic cells into iPSCs and then differentiate them into another cell type.

## First Generation CRISPRa Systems

First generation CRISPRa systems consist of two components: dCas9 fused with transactivator and sgRNA (Figure 1B). Whereas the design of sgRNA for knockout demands targeting on exons vital for protein function or structure, sgRNA for transactivation purposes should be complementary to upstream regions, proximal to a promoter.

The earliest CRISPR activators were based on direct dCas9 fusion with such transactivators as VP64, p65, and p300 (Gilbert et al., 2013; Hilton et al., 2015). VP64 is a tetramer of VP16 – a well-characterized transcription activator from the herpes simplex virus. Although both C-terminal and N-terminal placement of the transactivation domain are acceptable for chimeric dCas9 production, N-terminal localization exhibited the best fold-induction and efficiency (Mali et al., 2013; Duellman et al., 2017). VP64 demonstrated a strong induction of activation, which appears to be sufficient for several types of transdifferentiation experiment. For example, dCas9-VP64 was used by Chakraborty et al. (2014) to activate the *Myod1* gene in fibroblasts in order to successfully reprogram them into myocytes. It reveals the potential of dCas9 to replace exogenous overexpression methods. As well as single gene targeting, multi-gene activation has been proved to be effective by Chavez et al. (2015), who stimulated neuronal differentiation of human iPSCs by applying dCas9-VP64 technology. A similar experiment was conducted by Black et al. (2016), but they used dCas9 with both N-terminal and C-terminal VP64 transactivation domains. Unlike ectopic expression, activation of endogenous genes was rapid and remained high even through 18 days in culture.

In further studies, different oligomers of VP16 (VP48, VP160, and VP192) have been used as activators (Cheng et al., 2013; Duellman et al., 2017; Haenfler et al., 2018). It seems obvious that the efficiency of activation should grow with multiplexing of the VP16 domain and, indeed, VP160 usually demonstrates greater efficiency than VP64, but actually there is no convincing evidence that this correlation is true for all VP(16)n activators in all circumstances; rather it depends much more on the biological context.

Another transactivation factor, p65, which is the activation domain of NF-kappa B factor, can also contribute to transcription initiation. Although p65 has turned out to be less effective than VP64 and has rarely been used on its own, it had become part of another powerful activating system called VPR (Chavez et al., 2015). The VPR system was formulated after the Supernova tagging (SunTag) and Synergistic Activation Mediator (SAM) systems (see later) and involves transactivation by different effectors, thus, like them, it is often considered as a second generation system, but, as the activators are bound directly to the dCas9, here we have allocated it to the first generation group. VPR consists of activation domains VP64-p65-Rta (Rta for «replication and transcription activator» from the Epstein–Barr virus) linked with each other by short linkers and fused in tandem to the dCas9 (Figure 1C). The activation potential of the VP64-p65-Rta domains is of a high order, although effectors with other linkage orders are still capable of transactivation. Compared to VP64, VPR shows up to a 320-fold improvement to the activation of single endogenous targets and 3700-fold improvement during multiplex activation (Chavez et al., 2015; Fang et al., 2019). Recently, Weltner et al. (2018) demonstrated that human fibroblasts can be reprogrammed into iPSCs using only CRISPRa via VPH – a modified VPR system, where the Rta domain was substituted with the HSF1 (heat shock factor 1) activator domain. Interestingly, in this study, the targets of activation were not only the “classical” reprogramming factors OCT4, SOX2, KLF4, C-MYC, LIN28A, and NANOG, but also EGA-enriched Alu-motif (EEA-motif), which is considered to be involved in the control of early embryonic transcriptional networks. Targeting the EEA-motif enhances greatly the CRISPRa reprogramming efficiency, thus it demonstrates the importance of regulatory elements as potential targets for cell reprogramming.

It is important to note that, in the first CRISPRa experiments, one gRNA per one gene targeting was used, but further investigations showed that the addition of multiple gRNAs enhanced the activation greatly. This is accurate for all systems based on recruiting activator domains (Maeder et al., 2013; Perez-Pinera et al., 2013; Chavez et al., 2015).

In contrast to previous activators, p300 is the catalytic core of human acetyltransferase and it works through acetylation of histone H3 lysine 27, which leads to activation of both proximal and distant gene enhancers (Rada-Iglesias et al., 2010; Delvecchio et al., 2013). In addition, p300 can promote nucleosome remodeling (Shrimp et al., 2018). The dCas9-p300 has an increased transactivation capacity relative to dCas9-VP64. Moreover, the p300 effector is capable of activating gene expression robustly through a single gRNA; interesting, that additional sgRNAs demonstrate no synergy (Hilton et al., 2015).

Another important biological process that has inspired dCas9-based systems is DNA-methylation. DNA-methylation has an important role in the epigenetic control of eukaryotic gene expression (Smith and Meissner, 2013). Hypermethylation of promoter leads to silencing of the gene, whereas hypomethylation is considered to be an indicator of a potentially active promoter. In cells, demethylation is achieved by inhibition of DNMT1 (DNA methyltransferase) which normally maintains methylation. In addition, demethylation can be achieved through oxidation of the methyl group by ten-eleven translocation dioxygenases (TET) with subsequent base excision repair (Wu and Zhang, 2014). In the light of this, Liu X. S. et al. (2016) chose dCas9-TET1 as a demethylation effector in their study, where they were able to activate the *BDNF* gene in neurons; furthermore, they showed, that targeted demethylation of the *MyoD* distal enhancer facilitated myogenic reprogramming of fibroblasts. Most likely, demethylation is sufficient for reprogramming in only a minority of cases, but nevertheless it can facilitate transactivation and increase the efficiency of reprogramming. For example, Baumann et al. (2019) showed that removal of DNA methylation was able to increase transactivation of the master gene *Sox1* thus breaking down cell identity barriers.

Although first generation systems, except VPR, are significantly less efficient than second generation systems, they have one important advantage – the small size of the transgene, which is more preferable if the capacity of the vector is limited (Ma et al., 2018; Lau et al., 2019).

## Second Generation CRISPRa Systems

Second generation systems consist of three components: dCas9, sgRNA, and effectors, multiple copies of which are recruited by special domains on the dCas9 or sgRNA. This type of construction is believed to increase the effect of the manipulation being performed whether it is activation, repression, epigenetic modifications and, etc. Here we describe a variety of existing systems and compare them according to their efficiency and specificity.

### Scaffold and Casilio

The first steps toward the development of second generation systems were made when it was observed that the crystal structure of the dCas9:gRNA:DNA complex has the tetraloop and stem-loop 2 on the sgRNA protruding outside of the Cas9-sgRNA complex (Nishimasu et al., 2014). Since they are free from interactions with Cas9 it is possible to add there protein-interacting RNA aptamers to facilitate the recruitment of effector domains to the Cas9 complex.

**FIGURE 2:**
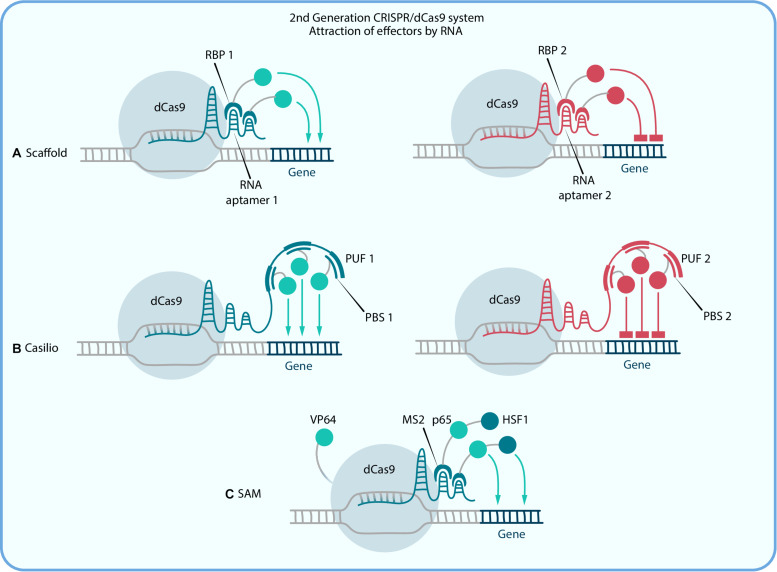
Second generation CRISPR/dCas9 systems, which activation or repression is amplified due to recruiting multiple copies of an effector by RNA. **(A)** Scaffold and **(B)** Casilio systems use RNA regions [aptamers in Scaffold and PUF-binding site (PBS) in Casilio] for simultaneous recruitment of different effectors to different sgRNAs by RNA binding proteins (RPB in Scaffold and PUF in Casilio). **(C)** in SAM RNA binding protein MS2 is fused with two activator domains, p65 and HSF1, and dCas9 is also fused with activator VP64.

The pioneer system of this kind is called Scaffold as it is based on scaffold RNA (scRNA) which is formed by introduction of hairpin aptamer domain, usually from MS2 bacteriophage, to the 3′ end of the sgRNA with a double-stranded linker between them for stability purposes (Zalatan et al., 2015). Aptamer-specific proteins (ex. MCP, PCP, and Com), fused with effectors, can bind to those sequences and thereby alter the target gene expression (Figure 2A). Different aptamers allow the recruitment of different effectors to the target site. To put it simply, in a Scaffold system, a single scRNA molecule encodes both information about the target locus and instructions about what regulatory function to execute at that locus. scRNA-recruitment of VP64 shows a greater expression activation than that for the direct dCas9-VP64 fusion protein (Zalatan et al., 2015). For example, in pioneer Zalatan et al. study the Scaffold strategy has been used to modulate a metabolic pathway in yeast cells, where distinct metabolites could be produced by simultaneous activation and repression of alternative enzymes with combinations of scRNAs. In mammalian cells, scRNAs recruiting VP64 have been used to activate *CXCR4*, and at the same time to repress β-1,4-N-acetyl-galactosaminyl transferase 1 (B4GALNT1) with the Krüppel-associated box domain (KRAB) repression domain (Dominguez et al., 2015). So, one of the main features of that system is the possibility of combining different effector domains and therefore performing complex gene regulation within one cell, and as a result, more efficient reprogramming can be performed than with the first generation techniques. The main limitation of this system is that the incorporation of three or more copies of aptamers in the scRNA sequence reduces its expression, which leads to a decrease of effector proteins that can be recruited. The Casilio system is an upgrade of Scaffold that was created to solve this problem. It relies on a combination of CRISPR-Cas9 and the Pumilio RNA-binding protein. Pumilio has an RNA-binding domain, PUF, that recognizes a specific 8-mer RNA sequence called the PUF-binding site (PBS; Figure 2B). Thus the Casilio system is very similar to Scaffold but includes dCas9, sgRNA with several PBSs and PUF-domains, fused with the effectors. The first main difference is that the linear architecture of the sgRNA-PBSs does not interfere with transcription, thereby allowing extensive multimerization of the PBSs, which is beneficial for the number of effector recruitments. In this way, Casilio improves on the sgRNA stability and potency (Cheng et al., 2016). Taghbalout et al. (2019) performed comparisons of different dCas9 activation systems based on TET1 demethylation and revealed that Casilio-based TET1 delivery outperforms both SunTag and Scaffold.

### Synergistic Activation Mediator

Further development of second generation Scaffold systems had led to the construction of dCas9-SAM, which is composed of three major components: chimeric dCas9-VP64, sgRNA with synthetic aptamers for MS2 recruitment and a chimeric MS2-p65-HSF1 activation helper protein (Figure 2C). This chimeric transactivator complex can be recruited to both the tetraloop and the stem-loop of the hairpin aptamer, which selectively binds dimerized MS2 bacteriophage coat proteins in mammalian cells (Konermann et al., 2014).

Synergistic activation mediator has the ability to upregulate genes greatly, as the recruited TFs work synergistically in order to activate the gene of interest. It has been shown that binding of the MS2-p65-HSF1 complex to the target region of the DNA could mediate transcriptional up-regulation more efficiently than does dCas9-VP64 fusion (Konermann et al., 2014). This technology has been successfully applied for the permanent elimination of HIV-1 latent reservoirs by precise identification of the enhancer region and reactivation of the HIV-1 provirus in HIV-1 latent cells (Zhang Y. et al., 2015). In addition, it was recently demonstrated that even single sgRNA-mediated SAM activation is sufficient for specific up-regulation of the entire 100 kb locus, C19MC (the microRNA cluster on chromosome 19; Mong et al., 2020). That study was dedicated to the investigation of C19MC’s role in epithelial-to-mesenchymal transition during implantation and it showed that miRNA, coded by this locus, increased the expression of *OCT4* and *FGF4*. Furthermore, it was found that activation of C19MC by SAM along with transfection of OCT3/4, SOX2, KLF4, and LIN28 increases the speed and efficiency of fibroblast reprogramming into iPSCs. Thus this study not only demonstrated SAM as a powerful activation tool but also indicates the potent application of CRISPRa technology both in fundamental developmental biology and cell reprogramming.

### Supernova Tagging System

In order to increase upregulation efficiency, another approach has also been applied. Instead of using a modified scaffold of guide RNA as the recruiters of effector proteins, researchers have tried to bind effectors to specifically designed peptide epitopes, fused to the dCas9. This approach is based on the fact that antibodies can bind to short peptide sequences with high affinity and specificity. Moreover, such “designed” epitopes are different from naturally occurring peptides in the cell, and that eliminates possible off-target binding.

**FIGURE 3:**
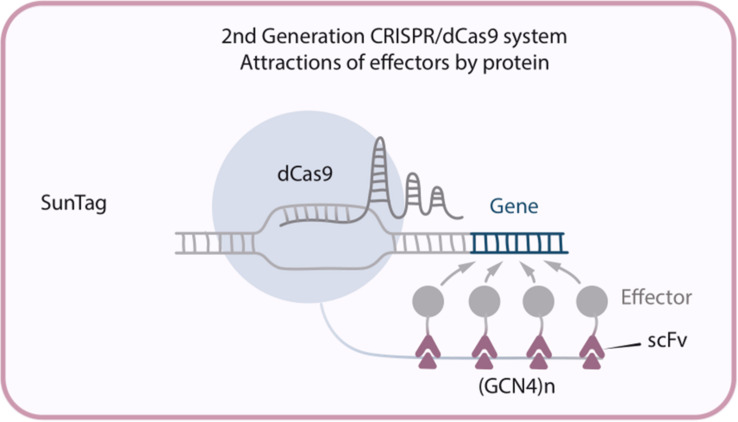
Second generation CRISPR/dCas9 system SunTag, in which multiple copies of GCN4 recruit multiple molecules of the chimeric scFv antibody-activator protein to the target site.

This new modification – dCas9-SunTag system – has been developed by Tanenbaum et al. (2014), and consists of three constructs: dCas9 fused with multiple copies of GCN4 peptide (this particular multi-peptide tag was called SunTag), an antibody single-chain variable fragment (scFv) fused with the activation domains (ex. VP64, p65-HSF1or p300), and sgRNA. (GCN4)n recruits n molecules of the chimeric scFv-activator protein to the target site, allowing significant amplification of the transactivation signal (Figure 3). To demonstrate the successful implementation of that new system dCas9-SunTag-VP64 was used to reactivate latent HIV-1 transcription in infected human T-cell lines and, what is more important, this took place without cytotoxicity, genotoxicity, or global T-cell activation (Ji et al., 2016). It is also possible to perform epigenetic modifications by fusing scFv with such effector domains like TET1 or DNA methyltransferase (DNMT). For example, it was shown that TET1-mediated CpG demethylation allows gene upregulation both in cell culture and in mouse fetuses (Morita et al., 2016) while dCas9-SunTag-DNMT3A increases CpG methylation at the HOXA5 locus in human embryonic kidney (HEK293T) cells (Huang et al., 2017).

The use of a single molecule of dCas9 that recruits multiple VP64 domains using SunTag has proved to be even more efficient, not only for single gene activation but also by making it possible to activate multiple genes simultaneously, allowing complex gene regulation. The SunTag system has been successfully applied for the generation of iPSCs, which requires the expression of four transcription factors (Takahashi and Yamanaka, 2006; Tanenbaum et al., 2014). Remodeling of the endogenous gene loci of Oct4 and Sox2 led to the induction of other pluripotent genes and, as a result, pluripotency induction in mouse fibroblasts (Liu P. et al., 2018). Further investigations applying large scale screening can help to identify proteins that promote induced pluripotency or cell differentiation. Summarizing the advantages of SunTag, we want to underline that combination of high efficiency and ability not only to switch on/off gene expression but also to moderate transcriptional activity by using a different number of effectors is what makes it quite outstanding and unique. As it was mentioned earlier, recruiting a different number of effectors at a certain point in the genome can be done by simple addition or removal of short GCN sequence in coding vector.

### Comparison of VPR, SAM, and SunTag Modifications

The VPR, SAM, and SunTag systems have shown a higher activation rate in comparison with more simple first generation dCas9-VP64 activator. SAM is the most consistent in delivering high levels of gene induction, although it has always remained within five-fold of either SunTag or VPR, neither of which is generally superior to the other. When it comes to multiple gene regulation there is a theory that the overall gene activation can be decreased, although no definitive evidence of this exists as yet (Konermann et al., 2014; Cheng et al., 2016). To test that theory, a set of experiments have been performed showing that, even within a complex activation scheme of 6 genes, all the systems showed similar levels of relative gene activation. Furthermore, differences in the basal gene expression of intact cells make all such comparisons irrelevant (Chavez et al., 2016).

Both the first and second generation systems (except dCas9-p300) are able to boost activation efficiency due to the recruitment of multiple sgRNAs to a single gene (Perez-Pinera et al., 2013; Chavez et al., 2016). Further improvements of dCas9-based activators, discovery of novel activation domains and exploration of epigenetic modifications can lead to more efficient expression activation of certain genes. As, for example, the integration of different modifications into a single one can help to reach higher levels of gene activation.

### Three-Component Repurposed Technology for Enhanced Expression

**FIGURE 4:**
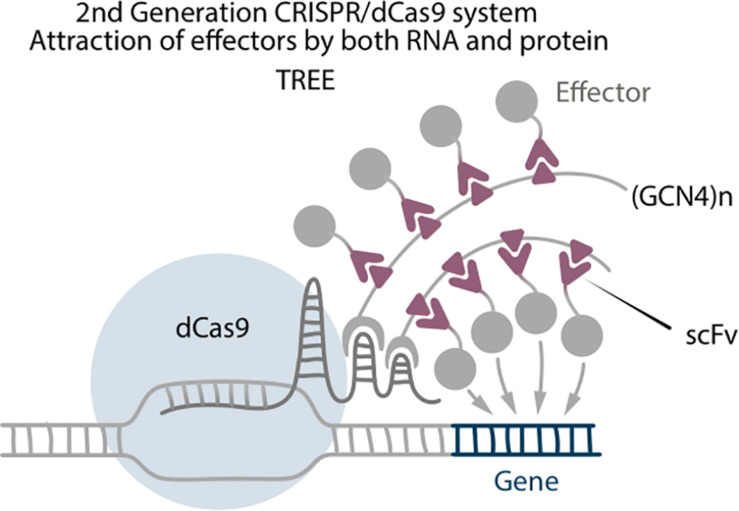
Second generation CRISPR/dCas9 system TREE, which is a combination of Scaffold and SunTag, where (GCN4)n tail binds with RNA aptamer by MS2 RNA binding protein.

Three-component repurposed technology for enhanced expression (TREE) is a strong transcriptional activation system, which combines attraction of effectors by RNA aptamers and by protein tags (Kunii et al., 2018). It consists of dCas9-VP64, sgRNA with two MS2 aptamers, SunTag fused with MS2 protein, and scFv-effector. In comparison with Scaffold systems, where the effector molecules are directly fused to the MS2 coat proteins, in the TREE system the effectors are connected with the scFv antibodies that bind to the GCN4 epitope as they do in SunTag systems (Figure 4). This results in a higher accumulation of effector molecules around the target site. Unfortunately, there are no TREE-mediated reprogramming experiments to date, but the system itself serves as an important example of how combination of different technologies can improve the outcome. Furthermore, enhancing accumulation of effectors is one of the main strategies to improve CRISPR-based editing (Kunii et al., 2020).

To sum up, transcriptional activation by CRISPRa is able to reveal complex molecular networks and cell interactions where gene knockout can be too dashing. This will help to understand the process of disease evolution and suggest further therapeutic strategies.

## CRISPRi Systems

So far we have been focused on activation strategies in particular due to their broad variety and applicability in reprogramming experiments. The CRISPR/Cas9 enhanced interference (CRISPRi) is also a very common tool based on the same principles as CRISPRa, but with alternative effector domains.

While transactivation is carried out mainly by the recruitment of transcription activators to gene promoters, suppression can be achieved either by direct physical blocking of RNA polymerases, by the hanging of special epigenetic markers or by condensing active euchromatin into silent heterochromatin. The very first CRISPR/Cas9-based suppressor of transcription was dCas9 itself. By binding to coding regions of DNA, it blocks RNA polymerase and transcript elongation (Qi et al., 2013). The dCas9:sgRNA complex has shown efficient repression of gene expression in *Escherichia coli*, but it turned out not to be sufficient enough to block eukaryotic RNA polymerases (Gilbert et al., 2014). Instead, improved effectiveness can be achieved by the fusion of transcriptional repressors to the dCas9 protein. A variety of modifications have been tested already, but the most efficient and reliable has turned out to be the KRAB, which promotes repression through the spreading of heterochromatin (Groner et al., 2010). Comparisons of different studies have shown that N-terminal fusion is preferable for more efficient suppression (La Russa and Qi, 2015). dCas9-KRAB provides strong and highly specific suppression of endogenous gene expression, especially if the sgRNA targets 50–100 bp regions downstream of the transcription start site (TSS; Gilbert et al., 2014). Importantly, some TSSs such as miRNA TSSs may be hard to reveal, even in fully sequenced genomes (Georgakilas et al., 2014). Moreover, TSSs of many genes can be different from each other not only in alternative transcripts but also in different cell types, therefore targeting the appropriate TSS is essential (Sandelin et al., 2007). On the other hand, sometimes, different transcripts share the same TSS, and in that case, targeting dCas9-KRAB could block the transcription of all of them at once (He et al., 2012). Investigating the epigenetic state of the targeting site can also make sense since the most effective binding of dCas9 occurs in the open chromatin areas determined by peaks of DNase I sensitivity (Kuscu et al., 2014).

It is known that methylation can be a repression tool and that the DNMTs DNMT3A and DNMT3B are responsible for this process. Thus chimeric dCas9-DNMT3A/B protein enables sequence-specific DNA methylation. dCas9-DNMT3A has been successfully used for gene silencing and, compared to dCas9-KRAB, provides more prolonged, but also less specific repression (Amabile et al., 2016). More robust suppression can be obtained by using both dCas9-KRAB and dCas9-DNMT3A or even dCas9-SunTag-DNMT3A systems (Huang et al., 2017; O’Geen et al., 2019).

One of the main advantages of CRISPRi is its low off-target activity. Firstly, the narrow binding site around the TSS restricts any modulations beyond it. Secondly, CRISPR activity is affected by any mismatches in the sgRNA:DNA pairing, which makes off-target binding too transient to have a great impact on the transcription in general (Gilbert et al., 2014; Wu et al., 2014).

## Large-Scale Genome Screening by CRISPR/dCas9

Despite the reach of the molecular toolkit that is provided by Cas9 and dCas9 especially, cell reprogramming and transdifferentiation is still a challenge. The main reason why changing cell fate is so difficult is a lack of knowledge of the molecular participants that take part in the development of every specific cell type. Investigations into complex gene networks and functional genomics should be the first step toward establishing accurate models of cell lineages, according to which it would be easier to perform rational reprogramming. This is where large-scale genome screenings can make a difference.

Large-scale genome screening has already revolutionized many fields of biology and medicine and has become applicable in a variety of research work. Screening can be a very powerful tool for the elucidation of gene function in the development of a disease. It helps to reveal the molecular players responsible for particular processes in the cell, and in discovering new genetic interactions in a large set of genes. Moreover, large-scale screening can be used to identify genes able to drive pluripotency induction. Investigations of genes responsible for drug resistance, or the identification of relevant molecular targets can be also be boosted with such techniques (Kampmann, 2017b). Not only the direct molecular targets of a drug can be found, but also the synergic ones for use in combinational therapy, as well as factors that are involved in drug-resistance (Acosta-Alvear et al., 2015).

As discussed earlier, CRISPR/Cas9 technology outperforms pre-CRISPR tools in many ways, starting from the simplicity of the constructions and their increased specificity (Shalem et al., 2013; Evers et al., 2016) through to their more affordable prices. Those advantages became a basis of use CRISPR/Cas9 systems in large-scale genome-wide screening, which demands a huge amount of different molecular components. Screening, in general, can be performed either by silencing the particular gene (loss-of-function) or by its activation (gain-of-function), and CRISPR/Cas9 can do both. Moreover, editing through epigenome alterations such as chromatin remodulation, DNA and histone modification and ncRNA relocation with CRISPR\dCas9 systems allow to conduct large-scale screenings for functional regulatory elements (Klann et al., 2017; Pulecio et al., 2017).

The key point in genome-wide screenings is the creation of sgRNA libraries targeting many genes at once. These libraries enable investigation of the functional properties of different genes and can reveal gene networks. sgRNA vector library is created by cloning a mixture of oligonucleotides. This library of barcoded sgRNAs is packaged into viral particles and transduced to mammalian cells at a low multiplicity of infection (MOI) with only one sgRNA per cell. Therefore it is possible to know which gene is activated or repressed in each cell. After the library has been constructed, the next step is to decide on the particular screening method. There are two main types of screening methods: arrayed and pooled. In the arrayed approach, each plate contains only one reagent, so only one genetic perturbation can be performed per well. This makes it expensive in that many different reagents have to be individually prepared. However, it does enable the investigation of a wide variety of cell phenotypes (Echeverri and Perrimon, 2006; Neumann et al., 2006). In pooled screening, the reagents are prepared in a mixture and diluted only by cell division or spontaneous degradation. This option is less expensive and more suitable for long-term culture hosting, although it is limited in the observed phenotypes when compared with the arrayed approach because it relies on the physical coupling of the phenotype to the genotype. Pooled screenings are not suitable for open reading frame (ORF) libraries due to the broad distribution of ORF sizes (Johannessen et al., 2010).

After modifications have been performed by either method the cells can be sorted and selected according to the presence of survival-enhancing perturbation (positive selection) or depletion (negative selection). This can be undertaken in many ways, but the most efficient are growth and sensitization/resistance screens. Unfortunately, not all biological questions can be translated into survival phenotypes, there being many other features that cannot be revealed by such an approach. To cope with this problem another type of cell sorting, named FACS, can be applied (Baron et al., 2019; Voronin et al., 2020). It is based on fluorescence activation with the subpopulations being isolated based on their fluorescence signals.

It is very important to keep in mind that preferable type of screen depends on target genes features. On the one hand, the cell cannot have a loss-of-function phenotype if a particular gene is not expressed, but a gain-of-function phenotype can be achieved in that case. This can reveal hidden networks in many biological processes. On the other hand, if overexpression is achieved for only one subunit of a protein complex, the gain-of-function phenotype may not be evident in such a case, whereas knockdown of one subunit may suppress the function of the whole complex and cause a loss-of-function phenotype.

The first tool applied for loss-of-function screenings was RNA interference (RNAi). RNAi is an RNA-guided biological mechanism of gene silencing by mRNA degradation. During RNAi, endogenous or exogenous double-stranded RNAs are processed first into short hairpin RNA (shRNA), and then into short single-stranded RNA – miRNA and siRNA, respectively. Short single-stranded RNA loads into the RISK complex, which uses its single-stranded version as a guide for substrate DNA targeting with its subsequent degradation. In RNAi-based screenings, synthetic precursors of shRNA or miRNA are used for silencing the genes of interest. Such technologies have shown efficient gene suppression in mammalian cell lines (Elbashir et al., 2001; Meister and Tuschl, 2004; Paddison et al., 2004; Chang et al., 2006). Although this method has made a significant contribution to the screening of many genes at a time (Berns et al., 2004; Boutros et al., 2004), it is hampered by incomplete repression and high off-target activity due to non-specific induction of miRNA-like effectors (Echeverri et al., 2006; Jackson and Linsley, 2010; Sigoillot et al., 2012).

Another strategy of loss-of-function screening is the induction of null mutations in tested genes. For this purpose, sequence-specific nucleases such as TALEN, ZFNs, and Cas9 are used (Gaj et al., 2013; Shalem et al., 2013), but CRISPR/Cas9 screen (CRISPRn) is the most popular because of its simplicity in use and modest cost, which is especially crucial for whole-genome screening. In contrast to RNAi, CRISPRn is not limited to RNA transcripts, so it can be used for establishing the role of any genomic region, not only the coding ones. Nonetheless, CRISPRn screening has several important limitations such as its inability to study essential genes and to perform sensitive analysis of regulatory elements and epigenetic modifications (Nagy and Kampmann, 2017). Another issue is that CRISPRn provides genetically heteromorphous cell populations, parts of which have normal non-knockout phenotype (González et al., 2014). Finally, the main principle of the technology – double-strand repair with NHEJ – may lead not only to frameshift indels, but also to in-frame indels and, as result, to the absence of knockout. The stochastic outcomes of CRISPRn due to unpredictable DNA repair can have a great impact on discovering genetic interaction maps when several genes are targeted simultaneously and all of them should be biallelically inactivated (Wong et al., 2016). It also should be mentioned that the delivery of Cas9 constructs can be inefficient in comparison with single-component shRNAs.

CRISPR interference screening can be effective where CRISPRn fails. Although it also produces polyclonal cell populations, analysis of multiple independent subclone lines has indicated that around 99% of them show complete loss of expression of the target gene (Mandegar et al., 2016). The principle of CRISPRi operation makes the level of repression tunable, and this allows scientists to reveal the functions of essential genes through partial knockdown (McInally et al., 2019). The off-target effect of CRISPRi is much lower than that of RNAi due to the narrower area around the TSS where CRISPRi can perform transcriptional regulation (Gilbert et al., 2014). No additional off-targets compared to CRISPRn have been revealed for CRISPRi (Mandegar et al., 2016). However, we have found conflicting information about which system is better for interference screening: in one study CRISPRn outperformed CRISPRi (Evers et al., 2016), whereas another showed significant knockdown dominance for CRISPRi (Mandegar et al., 2016), although both methods may derive complementary results since each of them has its own sources of false-positive results (Munoz et al., 2016; Rosenbluh et al., 2017). Thus new, more detailed research and comparisons need to be performed.

Gain-of-function screenings were firstly performed by the expression of ORFs from DNA vectors, which sometimes lead to overexpression beyond physiological levels, and are affected by unpredictable endogenous regulation. ORF can express mutant forms of proteins that differ from the host-cell phenotype and therefore enable screenings for point mutations in a given protein of interest (Berger et al., 2016). The effectiveness of first generation CRISPR/dCas9 tools with only one effector domain fused to the dCas9 were not sufficient to perform large-scale genome screening (Gilbert et al., 2013; Konermann et al., 2013). However, second generation systems have shown more promising results by recruiting multiple effectors and therefore improving upregulation activity. Furthermore, second generation systems provide robust activation via single sgRNA, and this is important as genetic screens generally require effective activation of the targeted gene with a single sgRNA. It has also helped to tackle the issues of inefficient bi-allelic mutagenesis in mammalian cells, which presented great challenges for the previous approaches. Large-scale screening has been performed utilizing various dCas9 modifications. With the help of the SAM system, screening for genes that confer resistance to an inhibitor of the proto-oncogene Ser/Thr kinase B-RAF (BRAF) has been carried out (Konermann et al., 2014). Screenings with the dCas9-SunTag system have also been performed for genes that modulate sensitivity to CTx-DTA (Cholera-diphtheria Toxin; Gilbert et al., 2014).

CRISPR-based screens have already demonstrated their efficiency in the reprogramming field (Shifrut et al., 2018; Yang et al., 2019; Yu et al., 2019). Liu Y. et al. (2018) went further: after the identification of murine neuronal-fate regulators, they tested whether pairwise activation was more efficient and if it was, for which factors. A combination of Ezh2 and Ngn1 was chosen for the derivation of neurons from ESCs by CRISPRa. Differentiated neurons showed electrophysiological functions very similar to native neurons. Furthermore, the authors performed transdifferentiation from embryonic fibroblasts to neurons using transgenic combinations of identified factors: Ngn1+Brn2, Ngn1+Ezh2, and Ngn1+Foxo1. Analysis of the patterns of expression and electrophysiological functions both demonstrated that transdifferentiated neurons also shared similarity to native neurons. Thus this study is a good example of how CRISPR-based systems can contribute both to screening and reprogramming. In addition, it demonstrates the importance of revealing the genetic interactions for increasing the efficiency of reprogramming. The authors also underlined that genetic interactions can depend on the level of gene expression, and therefore, that using precisely tunable CRISPRa/i, is a better screening strategy than the more robust transgenic or RNAi approaches.

Even though the direct fusion of effectors enables only one type of upregulation to be performed within one cell, Zalatan et al. (2015) tried to combine different types of effectors by using scRNA as has already been described above. Combining the CRISPRi/a approaches with the recruitment of different effectors for targeted mutagenesis or epigenome editing will enhance the efficiency of large-scale genome screenings. Pooled screens performed in human iPSC-derived cell types (Mandegar et al., 2016) may lead to the discovery of new therapeutic targets and contribute to the cure of severe diseases, such as neurodegenerative diseases (Kampmann, 2017a).

In order to investigate synergy between several genes, a new multi-functional genome-wide CRISPR screening system (MAGIC) has been designed recently (Lian et al., 2019). It combines CRISPRn, CRISPRa, and CRISPRi technologies for revealing interactions among the overexpression, repression, and deletion of different targets. Transfection of the host strain with pooled libraries of sgRNA-loaded plasmids and subsequent analysis by next-generation sequencing reveals the synergistic interactions of such targets. Although MAGIC has been successful in mapping the furfural resistance genes in yeasts, no experiments in other organisms have yet been reported. Nevertheless, this technology demonstrated the importance of a comprehensive analysis of gene networks and should not be ignored in future studies.

### Single-Cell CRISPR Screening

Single-cell RNA-seq analysis has been developed in order to provide a complete genome-wide description of the heterogeneity in cell populations (Shapiro et al., 2013; Klein et al., 2015). The combination of CRISPR based screenings with single-cell RNA-seq is the next step in elucidating gene functions on a large scale. Pooled screenings rely mostly on average phenotype readouts of the cell population, making it impossible to distinguish distinct perturbations that cause similar responses. Implementation of CRISPR/Cas9 pooled screening technologies along with single-cell RNA-seq allows profiling of the perturbations of all the target genes in each cell. Functional elucidation of multiple factors and their interactions is also possible, for example for the mammalian regulatory circuits of innate immunity (Jaitin et al., 2016). This method has evolved recently under many independent names: Perturb-seq (Adamson et al., 2016), CRISP-seq (Jaitin et al., 2016), or CROP-seq (Datlinger et al., 2017). All of these help to overcome research challenges such as the heterogeneity of cell cultures and the need for performing large-scale analysis in a more detailed manner (Junker and van Oudenaarden, 2014).

The key point in single-cell CRISPR screening is the modification of the lentivirus vector (that delivers the sgRNA) in such a way as to make identification of the sgRNA easier in a single cell from deep sequencing of the mRNAs (Lanning and Vakoc, 2017). Modifications can be in the form of recording a cassette of gRNA expression at the end of the puromycin resistance gene (which allows to detect the gRNA sequence when analyzing the 3′ expression after poly-A enrichment) or in the form of adding a special barcode sequence at the 3′-end of the gRNA, which allow identification of the gRNA sequence when using specially designed kits for scRNA-seq. The main challenge for the single-cell CRISPR screening method is analyzing a lot of noisy and partial data, which requires filtering and normalizing (Brennecke et al., 2013; Lun et al., 2016). Hopefully, many attempts to deal with such limitations are being performed. For example, there exists a versatile pipeline named MUSIC, which has been developed to run complex data aggregation, analysis, and visualization processes (Duan et al., 2019). Despite all the advantages of CRISPR single-cell screening coming from focusing on the regulation of only a single gene at a time, there are some drawbacks. Since targeting sgRNAs are introduced by lentiviral constructions at a low MOI this approach makes it difficult to follow *cis*-regulatory pathways as there are millions of candidate regulatory elements and ∼20,000 regulated genes in the human genome. Fortunately, a new expression quantitative trait locus (eQTL)-inspired framework has been introduced recently to address this problem (Gasperini et al., 2019).

## Discussion

From the multiplicity of CRISPR bioengineering systems arise a question of which one is better for a particular experiment. Although we had already compared some systems earlier in the article, here we want to generalize the pros and cons of each one of them. As it was mentioned earlier, the main difference between CRISPRn and CRISPRa/i systems is that the first one operates with nucleotide sequence while others modulate the efficiency of transcription and RNA level without performing alterations in DNA. Thus mutations created by CRISPR/Cas9 are permanent, which is suitable for reprogramming into mature somatic cells if they are differentiated terminally. In other cases, an induced mutation can complicate further differentiation and proliferation of obtained cells. Cell reprogramming with CRISPRa/i systems seems to be more flexible. It can be both permanent, if all components of the system are integrated into the genome and expressed constantly, and transient, when non-integrating vectors or inducible promoters are used. Ability to be switched on/off and also a vast range of possible modifications allows CRISPRa/i systems to outperform direct CRISPR/Cas9-mediated DNA editing in the reprogramming field.

### Limitations and Technical Issues of CRISPR/dCas9 Systems

Like others CRISPR-based systems, CRISPR/dCas9 has concerns, caused by off-target activity (Zhang X.-H. et al., 2015; Schaefer et al., 2017), although data had shown that dCas9-mediated alterations, based on deactivated Cas9 protein, are highly specific and, in some cases, even more specific than standart Cas9 (Qi et al., 2013; Gilbert et al., 2014; Wu et al., 2014; Thakore et al., 2015; Pulecio et al., 2017). Nevertheless, possible off-target effects should be taken into account. Non-specific transcription activation/repression can cause altered non-target gene expression and, as a result, disruption of dependent gene cascades, which is particularly dangerous for reprogramming into iPSC due to their high sensibility.

As far as off-target effects and efficient gene targeting both depend on sgRNA sequence, optimal design of sgRNA is necessary. Target sites are easily identified by bioinformatic tools or by scanning for 18–23 bp regions adjacent to a PAM site. Then it comes to identifying the on-target (efficiency) and off-target (specificity) scores in order to choose the most appropriate sgRNA. Many sgRNA design tools have been developed and they provide a choice from hundreds of reference genomes, selected PAM sites, and target sites while offering predictions of gRNA efficiencies (based on multiple scoring algorithms), as well as the probability of off-target effects (Haeussler et al., 2016). Importantly, the rules for optimal design of sgRNAs are nearly the same across different CRISPR systems (Chavez et al., 2016), therefore further improvements of sgRNA design and dCas9 constructs can help to overcome current limitations. Modern gRNA design tools for CRISPRi and CRISPRa, such as CHOPCHOP v3, consider positional factors such as nucleosome positioning, sequence features, and other factors in order to increase the efficiency of upregulation (Labun et al., 2019). The ability to recruit multiple effectors to one scaffold sgRNA enables bifunctional CRISPRa/i upregulation with only one dCas9 protein, thus decreasing the toxicity of its expression in human cells (Zalatan et al., 2015; Zhang and Voigt, 2018).

The specificity of the (d)Cas9 also contributes much to the process. More efficient variants of wild-type *Streptococcus pyogenes* Cas9 (SpCas9) have been designed recently, although they have not yet proved their efficiency in human cells (Lee et al., 2018). Different forms of Cas9 enzymes from various bacterial species will also allow to target different PAM sequences, expending the number of potent target sequences (Esvelt et al., 2013). Moreover, it will enable the combination of functional domains and therefore complex gene regulation in a single cell, a situation that presents many opportunities for uncovering sophisticated regulatory networks.

Another important technical aspect of cell reprogramming using CRISPR tools is sgRNA expression in the cell, which depends on the promoter. In mammalian cells, it can be either Pol III U6 promoter, with high fidelity, or the Pol II promoter offering increased processivity. Currently, most sgRNA expression relies on polymerase III promoters as they are constitutive, but in that case, the transcribed RNAs have short half-lives (Alic et al., 2007). Using polymerase II promoters could enable the production of multiple sgRNAs from a single transcript and therefore offer complex control over cell behavior. The main problem is the rapid export of most polymerase II transcripts to the cytoplasm, but this can be prevented by removing the 5′ cap and 3′ tail (Kruger et al., 1982), or including additional sequences into the intron (Kim, 2005). The problem with multiplexing the sgRNA to increase the scale of reprogramming or screening is that it can result in retroactivity, when different sgRNAs compete for available dCas9 proteins, as this alters the efficiency (Zhang and Voigt, 2018).

Editing with dCas9 has its own specific considerations. Since not only gene promoters can be targets of dCas9, but also regulatory elements, targeting them can influence the expression of many other genes, dependent on interaction with the target. For example, it was shown that one enhancer can regulate multiple genes (Gasperini et al., 2019), so its activation will probably result in the expression of all dependent genes. Thus, meticulously examine potential non-coding targets is required to avoid unwanted effects.

Cell differentiation is a complicated process, during which different genes cooperate to define future cell phenotype. The consequences of gene interactions are not limited by the presence or absence of their protein or RNA products, but the level and longevity of expression also matter a lot. For example, mammalian testicular formation demands the expression of SRY in a narrow time window and on a sufficient level, otherwise, bipotential genital ridge fails to obtain the male phenotype (Kashimada and Koopman, 2010). Thereby the ability to control the working time of the CRISPR/Cas9 is crucial in cell reprogramming due to its correlation with cell fate; it also prevents off-target effects caused by the prolonged activity of dCas9.

For *in vivo* manipulations it is necessary to have both the effector and the switch-off system available at once, to avoid consequences of uncontrolled modifications (Bondy-Denomy, 2018; Nakamura et al., 2019). Therefore various controlling systems have been developed recently, such as numerous methods for system deactivation, inducible promoters, and anti-CRISPR inhibitors. For example, Balboa et al. (2015) fused an inducible destabilization domain (DHFR from *E. coli*) to dCas9 to quickly depress dCas9-mediated activation, which enabled differentiation of human pluripotent stem cells into pancreatic progenitor cells. Cas9 protein can be also turned on selectively by using light- or chemically inducible promoters (Polstein and Gersbach, 2015). Another set of methods for transcriptional control includes programmable synthetic genetic circuits (Weinberg et al., 2017).

Working with cell populations has several drawbacks such as heterogeneity of gene expression between cells of the same type (Pandelakis et al., 2020). As well as cells from different tissues and organs have a different response to the same transcriptional regulator depending on their microenvironment, cell interactions, and cellular state. The genetic engineering construct itself can behave differently in the case of the variability of 3D genome structure around the target gene, TFs interactions, and epigenetic marks (Pichon et al., 2018). This should be considered when the same principles of reprogramming are applied to different cell types.

### Targets of Cell Reprogramming

While a majority of CRISPRa/i studies have focused on investigating genes, it is important to remember that the phenotype is a result of complex well-coordinated interactions of many genes and their regulatory elements such as promoters, enhancers, silencers and non-coding RNAs (ncRNAs); and that all these interactions are influenced by epigenetic modifications of DNA, nucleosome localization and the state of the chromatin. Thus the search for reprogramming factors should not be restricted to transactivators. We have already mentioned several works, where a combination of transactivation and epigenetic modification has greatly increased reprogramming efficiency (dos Santos et al., 2014; Wang Y. et al., 2017). As for ncRNA, there are different types of ncRNA, which demand different strategies of identification. miRNAs are coded by genes, so for their screening, the same rules as for protein-coding genes can be applied. For example, Panganiban et al. (2019) uncovered multiple suppressors of endoplasmic reticulum stress via CRISPRn screening, among which was miRNA-124, and indeed in a further study miRNA-124 has shown to provide protection from arsenic-induced endoplasmic reticulum stress and a potential therapeutic target to counteract arsenic exposure (Park et al., 2020).

Another important group of regulators includes long non-coding RNA (lncRNA). Since the biological functions are established for only a very small proportion of lncRNAs, CRISPR-based screenings can contribute greatly to this field. Although CRISPRn and CRISPRa/i screens have already helped to identify lncRNAs involved in some biological processes (Liu S. J. et al., 2016; Joung et al., 2017; Liu et al., 2020), there are several issues that make such screening studies complicated. First of all, lncRNAs are distributed throughout the genome, including both the intra- and intergenic regions; moreover, intragenic localization can be intronic, sense, and antisense exonic, or exonicoverlapping with a part of a sense gene. lncRNAs can be produced from their own promoters or from promoters shared with other genes (Awwad, 2019). Taken together, these facts show that it could be challenging to apply the CRISPRn screening approach without the risk of disrupting non-target “host” genes or “common” promoters (Liu et al., 2020). Furthermore, small indels, performed by Cas9, do not generally abolish the biological activity since the transcription of lncRNAs, by itself, can have functional consequences (Kornienko et al., 2013). This limitation is also common for RNAi based screening, but CRISPRi overcomes it by suppressing transcription directly. Speaking of RNAi, since its machinery operates mostly in the cytoplasm, it can be difficult for it to effectively target lncRNAs (Fatica and Bozzoni, 2013). The nuclear localization of CRISPRa/i and its non-DNA-disruption principle of operation make it more suitable for lncRNA screening than CRISPRn or RNAi.

### Delivery of CRISPR/dCas9 Components Into Cell

Besides considering all the above points on reprogramming constructs, the delivery of them to the host-cell can also make a difference. Most often, lentiviral vectors are used for the delivery *in vitro* of CRISPR/Cas9 components. Lentiviruses have a packing capacity of around 8 kb, transduce dividing and non-dividing cells, and can be pseudotyped with other viral proteins in order to alter their tropism (Lino et al., 2018). Additionally, they provide a highly efficient expression of the delivered transgenes due to their integration into the host genome. This “advantage” can also be a disadvantage at the same time: non-specific integration risks causing severe mutations, even though lentiviruses tend to integrate away from the cellular promoters (Vannucci et al., 2013). This risk is acceptable for *in vitro* reprogramming experiments to some extent, but is poorly-tolerated in *in vivo* strategies and especially when it comes to clinical applications of the engineered tissues.

The application of adeno-associated viruses (AAVs) is mainly episomal or involves integration into the strictly adeno-associated virus integration site 1 (AAVS1) without any side effects, and helps to avoid insertional mutagenesis (Kotin et al., 1990). Thus, these are more promising vectors for clinical-oriented studies. In fact, AAVs have already been approved for human clinical trials (Lau and Suh, 2017). Like lentivirus, AAV has a wide-range tropism due to the multiple available serotypes. Due mostly to their episomal form, AAVs are inferior in expression efficiency to lentiviral vectors, but their main disadvantage is the low packaging capacity of around only 5 kb, thus more than one vector is needed to deliver all the CRISPR/Cas9 components. Addressing this issue, several groups have designed compact systems based on small Cas9 homologs like Staphylococcus aureus Cas9 (SaCas9; Nishimasu et al., 2015; Ma et al., 2018; Lau et al., 2019).

In the case of the *in vivo* performance of reprogramming experiments the number of vectors and size of the transgene is of particular importance – the smaller the better. In this regard, systems that can produce both editing and control of gene expression are the most encouraging. In this context Scaffold CRISPRa/i systems are the most promising. Surprisingly, Cas9 is an option too, because it has been shown that targeting with gRNAs containing 15–16 nt results in a drastic reduction in nuclease activity (Fu et al., 2014). Further studies have demonstrated that the use of chimeric Cas9-transactivator in combination with 14–16 nt sgRNA activated gene expression successfully along with no significant increase of off-target effects (Dahlman et al., 2015; Kiani et al., 2015). Thus a dual-action two-component Cas9-effector system may present a solution when both editing and expression remodeling are needed within a minimal transgene capacity vector. Besides the two aforementioned virus-based vector systems, there are many other different approaches for delivering transgenes into cells (Lino et al., 2018), but since they are not currently popular within reprogramming studies, we will not discuss them in this paper.

## Conclusion

Manipulating both DNA and RNA during cell reprogramming is an attractive perspective, especially when it comes to the treatment of genetic disorders. The production of different sorts of healthy cells from mutant somatic cells is one of the main goals for modern regenerative medicine. Today the first step to achieve this goal can be a combination of Cas9-mediated gene engineering and the dCas9-mediated control of gene expression. Even though its advantages outweigh the drawbacks for the moment, many improvements are still required. A combination of cheapness, simplicity, specificity, and high efficiency already makes CRISPR/Cas9 based systems an indispensable tool for all sorts of experiments from precise point activation to whole-genome screens. The speed of evolution of CRISPR/Cas9 technology gives us hope that in a few decades we will be able to diagnose and treat the most severe diseases and produce all variety of patient-specific tissues and organs for regenerative medicine. This is the time for fundamental science to cooperate closely with medicine when scientists and clinicians working side by side can make a huge contribution to the wellbeing of society.

## Author Contributions

KS and VO wrote and edited the manuscript. ED developed the concepts and edited the manuscript. All authors contributed to the writing, reading, and approval of the final manuscript.

## Conflict of Interest

The authors declare that the research was conducted in the absence of any commercial or financial relationships that could be construed as a potential conflict of interest.
